# Phase 1–3 of the cross‐cultural development of an EORTC questionnaire for the assessment of sexual health in cancer patients: the EORTC SHQ‐22

**DOI:** 10.1002/cam4.1338

**Published:** 2018-02-13

**Authors:** Anne Sophie Oberguggenberger, Eva Nagele, Elisabeth C. Inwald, Krzysztof Tomaszewski, Anne Lanceley, Andy Nordin, Carien L. Creutzberg, Karin Kuljanic, Dimitrios Kardamakis, Claudia Schmalz, Juan Arraras, Anna Costantini, Thierry Almont, Chie Wei‐Chu, Sara Dehandschutter, Zoe Winters, Elfriede Greimel, the EORTC Quality of Life Group

**Affiliations:** ^1^ Department of Psychiatry, Psychotherapy and Psychosomatics Medical University of Innsbruck Innsbruck Austria; ^2^ Department of Obstetrics and Gynecology Medical University of Graz Graz Austria; ^3^ Department of Gynecology and Obstetrics University Medical Center Regensburg Regensburg Germany; ^4^ Health Outcomes Research Unit Department of Gerontology, Geriatrics, and Social Work Faculty of Education Ignatianum Academy Krakow Poland; ^5^ Department of Women's Cancer University College London UK; ^6^ East Kent Hospital Kent UK; ^7^ Department of Clinical Oncology Leiden University Medical Center Leiden The Netherlands; ^8^ Department of Gynaecology and Obstetrics University Hospital Centre Rijeka Rijeka Croatia; ^9^ Department of Radiation Oncology University of Patras Medical School Patras Greece; ^10^ Department of Radiotherapy Christian‐Albrechts‐University Hospital Kiel Kiel Germany; ^11^ Oncology Department Hospital of Navarre Pamplona Spain; ^12^ Psychoncology Unit Sant'Andrea Hospital Sapienza University Rome Italy; ^13^ Institute Universitaire du Cancer Toulouse France; ^14^ Institute of Preventive Medicine National Taiwan University Taipei City Taiwan; ^15^ University Hospitals Leuven Leuven Belgium; ^16^ School of Clinical Sciences Southmead Hospital University of Bristol Bristol UK

**Keywords:** Cancer, patient‐reported outcomes, quality of life, sexual health, survivor

## Abstract

To develop and pretest an European Organization for the Research and Treatment of Cancer Sexual Health Questionnaire (EORTC SHQ‐22) for the assessment of physical, psychological, and social aspects of sexual health (SH) in male and female cancer patients and survivors. Questionnaire construction started with creating a list of relevant SH issues based on a comprehensive literature review. Issues were subsequently evaluated for relevance and prioritization by 78 healthcare professionals (HCP) and 107 patients from 12 countries during in‐depth interviews (phase 1). Extracted issues were operationalized into items (phase 2). Phase 3 focused on pretesting the preliminary questionnaire in a cross‐cultural patient sample (*n* = 171) using debriefing interviews. Psychometric properties were preliminary determined using a principal component analysis and Cronbach's alpha. We derived 53 relevant SH issues from the literature. Based on HCP and patient interviews, 22 of these 53 issues were selected and operationalized into items. Testing the preliminary 22‐item short questionnaire resulted in a change of wording in five items and two communication‐related items; no items were removed. Preliminary psychometric analysis revealed a two‐factor solution and 11 single items; both scales showed good reliability indicated by a Cronbach's alpha of 0.87 (sexual satisfaction) and 0.82 (sexual pain). Cross‐cultural pretesting of the preliminary EORTC SH questionnaire has indicated excellent applicability, patient acceptance, and comprehensiveness as well as good psychometric properties. The final development phase, that is psychometric validation (phase four) including large‐scale, cross‐cultural field testing of the EORTC SHQ‐22, has commenced.

## Introduction

Cancer diagnosis and related treatments are well known to induce serious adverse effects regarding patients’ sexuality 1, 2, 3, 4. Aggressive cancer treatment strategies can adversely impact on a patient's physical functioning, for example reduced sexual desire, orgasmic problems, erectile dysfunction (males), and dyspareunia (females) 5 as well as body perception and emotional stability. In addition, cancer can trigger psychosexual and socio‐behavioral problems including feelings of sexual unattractiveness, alterations to the patient's sexual self‐conception, or reproductive concerns 6, 7, 8, 9. Unlike many other consequences of a cancer diagnosis, sexual impairments are not restricted to the treatment phase but highly persist in the survivorship period 10. As a consequence, patients report notable reductions in quality of life (QOL) and a disruption of return to “normal” life after cancer treatment 11, 12.

In order to comprehensively understand and adequately determine the impact of cancer on a patient's SH, it is crucial to conceptualize SH as a multidimensional construct, not only restricted to physical–functional aspects of sexuality but also comprising a psychosexual and socio‐behavioral component 13, 14. This is reflected by the WHO definition of SH as a state of physical, emotional, mental, and social well‐being related to sexuality 13.

Currently, a well‐validated patient‐reported outcome (PRO) measure applicable to female and male cancer patients that meets these requirements of a multidimensional understanding of SH is lacking 15, 16, 17, 18. Available instruments are either group‐specific, have not been validated for use in cancer care, have limited multicultural applicability, or do not cover the full range of bio‐psycho‐social sexual issues.

Thus, the EORTC Quality of Life Group (QLG) decided to develop a comprehensive EORTC SH questionnaire relevant to all patients with cancer. The EORTC QLG aims to develop reliable, high‐quality PRO instruments for measuring the multidimensional aspects of QOL in patients with cancer based on questionnaire development guidelines 19. These PRO instruments including the EORTC QLQ‐C30 and its modules can be used as a QOL outcome measure in clinical trials or for the purpose of monitoring in daily clinical practice 20.

The objective of our study was to develop an EORTC SH questionnaire applicable to male and female cancer patients at different treatment stages and in the survivorship phase that reflects SH as a multidimensional construct. In this article, we present the results of the development phases 1**–**3 of the EORTC SHQ‐22.

## Patients and Method

The study protocol was approved by the local ethical committees according to the national requirements.

### Phase 1—Generation of QOL issues

#### Literature search

The literature was exhaustively searched using PubMed targeting on SH issues in patients/survivors with any cancer diagnosis. The search covered original, qualitative, English language research papers published in peer‐reviewed journals from year 1993. We used an inclusive search strategy for sexual health and sexuality‐related issues in patients and survivors with any cancer diagnosis. The following combinations of keywords were used: neoplasms [mesh] OR neoplas*[tw] OR tumor [tw] OR tumors [tw] OR tumou*[tw] OR cancer*[tw] OR carcinom*[tw] OR oncolog*[tw] AND Sexuality”[Mesh]) OR (sexual function) OR (sexual function[All Fields]) OR (sexual function[tiab]) OR (sexual dysfunction) OR (sexual dysfunction [All Fields]) or (sexual dysfunction [tiab]). Available SH self‐report measures, derived from the Patient‐Reported Outcome Quality of Life Instruments Database (PROQOLID), were reviewed 21. We excluded quantitative studies of sexual health issues indicated by total or subscale scores (no analysis by thematic content).

#### Interviews with patients and healthcare providers (HCP)

Extracted issues were evaluated in semistructured interviews with HCP. HCP were eligible if they had at least 6 months experience in cancer care, were experienced with SH in oncology, and were fluent in the language of the questionnaire provided. Patient eligibility criteria included the following: histologically confirmed diagnosis of cancer, any cancer site and stage, any time point on treatment/survivorship pathway, no cognitive impairments, mother tongue of the questionnaire provided, 18 years of age or above, and written informed consent. Both HCP and cancer patients/survivors rated the issue list for relevance on a 4‐point Likert scale (0 = “not relevant at all” to 3 = “very relevant”) and prioritized 50% of issues (dichotomously) in order to determine their relative importance. Patients were invited to give feedback and suggest any other relevant issues not appearing on the list in order to determine breath of coverage.

#### Issue selection procedure—phase 1

Based on the results of the literature search and the evaluation by patients and HCP, SH issues were defined relevant and selected for further inclusion in the preliminary questionnaire according to the following predefined criteria in phase 1:

1. Mean score in HCP ≥ 2.
2. Priority rating HCP > 30%.
3. Mean score in patients ≥ 2.
4. Priority rating patients > 30%.

Issues that met 3 or 4 criteria were retained on the list; issues meeting 2 criteria or less were deleted. New issues generated by open feedback were included if mentioned by a third of the participants at least.

### Phase 2—Construction of the item list

Retained issues were operationalized into items (4‐point Likert scale), wording, and time frame (during the last 4 weeks) compatible with the EORTC QLQ‐C30 and its modules. The EORTC item library 22 was checked for relevant items; issues lacking an appropriate item in the item library were newly formulated. Item translation included a standardized forward–backward procedure 19.

### Phase 3—Pretesting of the provisional questionnaire

In phase 3, the provisional questionnaire was pretested. Cancer patients and survivors representing the target population completed the preliminary EORTC SHQ‐22, followed by a structured interview with the researcher focusing on item wording, intrusiveness, comprehension, and comprehensiveness. Phase 3 patient eligibility criteria were idem to phase 1.

#### Issue selection procedure—phase 3

Criteria for item selection in phase 3 were as follows:

1. Mean score > 1.5,
2. Prevalence of scores 3 or 4 > 50%,
3. Range > 2 points,
4. Each of the four response categories of an item (not at all, a little, quite a bit, very much) was selected by at least 10% of the patients,
5. Less than 10% missing responses to the item and no significant concerns raised by participants. Items that were identified as troublesome or difficult to understand were considered for rephrasing or rewording.

#### Patient recruitment

In the collaborating centers, patient recruitment was conducted in accordance with requirements of the local ethical committees. Patients were approached by the collaborator or his or her research assistant within the clinical routine, invited for study participation, and provided written informed consent (in case of participation). Subsequently, patients completed the issue list and the semistructured interview (given previously) in phase 1; in phase 3, they completed the preliminary questionnaire and the debriefing interview. HCP were recruited directly by the collaborators in his or her research team and underwent the same assessment as patients did in phases 1 and 3.

#### Cross‐cultural and cross‐lingual questionnaire development

In order to guarantee a cross‐cultural and cross‐lingual applicability and validity of the questionnaire, the EORTC QoL group recommends a balanced distribution of countries/languages involved in the development process. To meet these requirements for the purpose of this study, we followed the EORTC QoL group questionnaire development guidelines indicating that “the construction process should include at least three languages and countries, to include one representing each of the following groupings: (a) English‐speaking countries; (b) Northern Europe; and (c) Southern Europe” (p. 4) 19 for phase 1; phase 3 is supposed to be conducted in a wider range of countries and regions including at least six countries from English‐speaking countries, northern, southern, and eastern Europe, and one non‐European country. This project was, hence, conducted by collaborators from Austria, Croatia, Germany, Italy, the Netherlands, Spain, Taiwan, UK, Belgium, Greece, Poland, and France.

#### Statistical analysis

We analyzed results descriptively using means, standard deviations, and percentages in phase 1. In order to perform preliminary analysis of the scale structure of the questionnaire in phase 3, we used an explorative principal component analysis with the oblique rotation method (promax rotation) for factor extraction; the optimal number of factors was determined using eigenvalues >1. Conditional items (i.e., items for females and males only) were excluded from the analysis. Scale reliability (internal consistency) was determined by means of Cronbach's alpha. SPSS 22 was used for the statistical data analysis 23.

## Results

**Figure 1:**
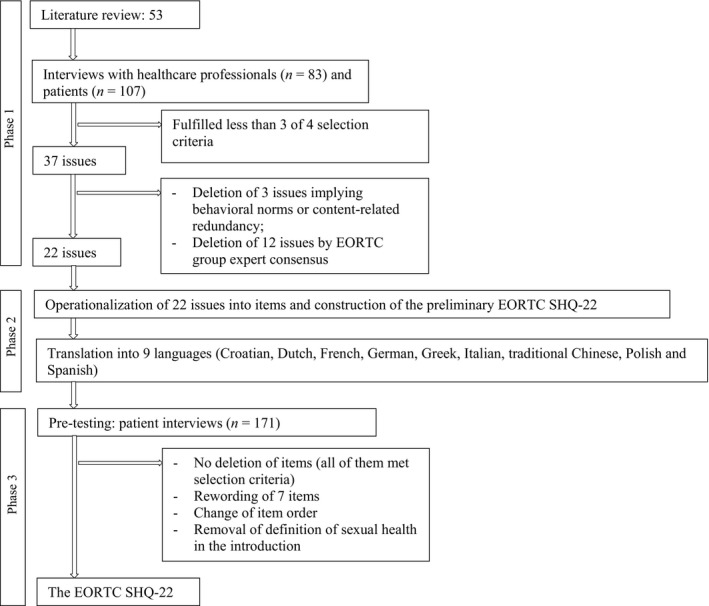
Process of issue generation and item selection across phase 1–3.

The entire process of issue generation and item selection across all phases is illustrated in Figure 1.

### Phase 1—Generation of QOL issues

#### Questionnaire structure

The questionnaire is designed and validated as a stand‐alone measure allowing its use independent from the EORTC QLQ‐C30 or together. This approach was agreed as the EORTC QLQ‐C30 does not address SH as a subdomain of QOL relevant to all patients with cancer, thereby preventing a targeted supplementation of the questionnaire which is the development model for other disease‐specific modules.

#### Literature review

Overall, 4518 PubMed records were screened by title and abstract, resulting in the exclusion of 3461 records. The remaining 989 manuscripts were screened; in‐depth review of 65 articles was finally conducted. The literature review identified a total of 53 issues relating to the sexual response cycle and side effects impacting on sexual activity (physical domain), relationship and intimacy (social domain), global SH (psychological domain), and sex‐specific issues as well as communication with HCP.

#### Interviews

A total of 107 patients with different cancer diagnoses and treatment stages from eight countries participated. About a fourth of patients were survivors, that is, had treatment more than 5 years previously, and were free from disease. Details on the clinical and sociodemographic patient data are shown in Tables 1 and 2. In total, an interdisciplinary, cross‐cultural sample of 83 HCP from 13 countries (Australia, Austria, Belgium, Germany, Hungary, Denmark, Italy, Norway, the Netherlands, Spain, Sweden, United Kingdom and Taiwan) was included. HCP had different professional backgrounds (radiation oncology *n* = 20; medical oncology *n* = 5; gynecology *n* = 18; and general surgery physicians *n* = 10; specialist nurses *n* = 4; psycho‐oncologists *n* = 16; and others *n* = 6) and were highly experienced with SH issues in oncology with more than half of the HCP having 10 or more years of clinical experience. A total of 56% of the HCP were females.

**Table 1:** Sociodemographic and sexual health‐related patient characteristics

	Phase 1 (*N* = 107)		Phase 3 (*N* = 171)	
	*n* a	%	*n*	%
Country				
Austria	12	11.2%	11	6.4%
Croatia	15	14.0%	15	8.7%
Germany	21	19.6%	19	11.1%
Italy	10	9.3%	9	5.2%
Netherlands	15	14.0%	25	14.6%
Spain	10	9.3%	15	8.7%
Taiwan	10	9.3%	4	2.3%
UK	13	12.1%	13	7.6%
Belgium	–	–	15	8.7%
Greece	–	–	10	5.8%
Poland	–	–	17	9.9%
France			13	7.6%
Sex				
Female	66	62%	101	59%
Male	41	38%	70	41%
Age				
Mean (SD)	55 (11) years		55 (13) years	
range	21–81 years		20–91 years	
Sexual partner^a^				
Yes	90	84%	143	84%
No	15	14%	26	15%
Education level				
Compulsory school education or less	24	22%	40	23%
Postcompulsory school education	47	44%	72	42%
University level	30	28%	59	35%

Current sexual partner.

**Table 2:** Clinical patient characteristics

	Phase 1 (*N* = 107)		Phase 3 (*N* = 171)	
	*n*	%	*n*	%
Cancer site^a^				
Breast	43	40%	52	30.4%
Colorectal	17	16%	13	7.6%
Head and Neck	14	13%	15	8.7%
Prostate	8	7%	23	13.4%
Lung	5	5%	9	5.2%
Cervical	4	4%	8	4.6%
Ovarian	4	4%	12	7%
Endometrial/Cervical	2	2%	8	4.6%
Other (anal, kidney, testicular, pancreas)	9	9%	23	13.4%
Other gynecological cancers	–	–	11	6.4%
Treatment status				
Active treatment	72	67%	121	71%
No active treatment	34	32%	49	29%
Treatment^b^				
Surgery	76	–	121	–
Radiation therapy	66	–	110	–
Chemotherapy	62	–	99	–
Antihormonal therapy	28	–	36	–
Others	5	–	18	–
Status of disease				
No evidence of disease	53	50%	43	26%
Newly diagnosed	17	16%	90	54%
Recurrence	11	10%	17	10%
Progression	10	9%	18	11%
Survivor	23	22%	16	10%
Time of treatment completion				
During the last 5 years	45	42%	73	43%
More than 5 years ago	9	8%	5	3%
Not completed yet	4	4%	5	3%
Missing	49	46%	88	51%

a Five patients had more than one cancer site. Only the current or main cancer sites are included in this table.

b Multiple answers were possible.

Based on quantitative and qualitative interview data, the list was reduced to 22 issues as follows: A total of 37 issues fulfilled at least three of four retention criteria. Of these, another 15 issues were deleted since implying behavioral norms such as frequency data or content‐related redundancy. Qualitative data did not generate additional issues. Table 3 presents the selection procedure of issues (i.e., physical, psychological, and social domain).

**Table 3:** Decision on issue deletion after phase 1 based on relevance and priority ratings of patients (*N* = 107) and HCP (*N* = 83)

Issue	Relevance ratings (0–3 points)		Priority for inclusion (yes–no)		Criteria
	HCP ratings Mean	Patient ratings Mean	HCP ratings *N* (%)	Patient ratings *N* (%)	Fulfilled
Frequency of sexual activity	1.81	1.68	30 (36)^c^	53 (50)^c^	2 of 4
Reasons for being sexually inactive	2.20^a^	1.57	39 (47)^c^	39 (36)^c^	3 of 4^d^
Satisfaction with the frequency of sexual activity	**2.26** a	**2.05** b	**48 (58)** c	**68 (64)** c	**4 of 4**
Importance of having an active sexual life	**2.28** a	**2.10** b	**40 (48)** c	**57 (53)** c	**4 of 4**
Level of hesitation to initiate sexual activities	1.81	1.47	21 (25)	35 (33)^c^	1 of 4
Frequency of sexual desire	1.76	1.76	18 (22)	45 (42)^c^	1 of 4
Level of desire	1.79	1.70	18 (22)	39 (36)^c^	1 of 4
Distress caused by decreased libido	**2.43** a	**1.61**	**55 (66)** c	**51 (48)** c	**3 of 4**
Satisfaction with frequency of sexual desire	2.02^a^	1.91	30 (36)^c^	55 (51)^c^	3 of 4^d^
Satisfaction with level of desire	2.16^a^	1.74	27 (33)^c^	47 (44)^c^	**3 of 4**
Frequency of sexual arousal	1.70	1.56	15 (18)	40 (37)^c^	1 of 4
Level of sexual arousal	1.85	1.52	13 (16)	36 (34)^c^	1 of 4
Satisfaction with level of sexual arousal	**2.38** a	**1.86**	**50 (60)** c	**60 (56)** c	**3 of 4**
Ability to achieve an orgasm	2.30^a^	1.86	43 (52)^c^	64 (61)^c^	3 of 4
Difficulty to reach an orgasm	2.15^a^	1.54	35 (42)^c^	47 (44)^c^	3 of 4
Satisfaction with the ability to orgasm	**2.48** a	**1.69**	**46 (55)** c	**53 (50)** c	**3 of 4**
Satisfaction with the frequency of orgasm	2.10^a^	1.58	31 (37)^c^	45 (42)^c^	3 of 4
Incontinence (urine/fecal) during foreplay or intercourse	**2.39** a	**0.87**	**55 (66)** c	**37 (35)** c	**3 of 4**
Hair loss (indirectly) affecting sexual response	1.72	0.93	25 (30)	39 (36)^c^	2 of 4
Fatigue/lack of energy affecting sex life	**2.45** a	**1.75**	**56 (67)** c	**66 (62)** c	**3 of 4**
Scarring/organ loss (indirectly) affecting sexual response/satisfaction	**2.42** a	**1.45**	**51 (61)** c	**50 (47)** c	**3 of 4**
Frequency of pain during/after sexual activity	2.54^a^	1.39	62 (75)^c^	53 (50)^c^	3 of 4^d^
Level of pain during/after sexual activity	**2.57** a	**1.26**	**57 (69)** c	**41 (38)** c	**3 of 4**
Change in amount of affection expressed	2.14^a^	1.71	30 (36)^c^	62 (58)^c^	3 of 4
The level of emotional intimacy	2.13^a^	2.10^b^	22 (27)	61 (57)^c^	3 of 4
Satisfaction with level of affection or intimacy	**2.31** a	**2.24** b	**55 (66)** c	**64 (60)** c	**4 of 4**
Fear that sex will be painful	**2.46** a	**1.06**	**59 (71)** c	**45 (42)** c	**3 of 4**
Fear of injury during intercourse	2.08^a^	0.87	31 (37)^c^	30 (28)	2 of 4
Fear harming the incision during intercourse	1.78	0.93	14 (17)	22 (21)	0 of 4
Satisfaction communication partner	**2.43** a	**2.35** b	**54 (65)** c	**73 (68)** c	**4 of 4**
Partner is afraid to touch. afraid to cause pain	**2.21** a	**1.54**	**37 (45)** c	**49 (46)** c	**3 of 4**
Experience of emotional distance from spouse	2.35^a^	1.40	46 (55)^c^	51 (48)^c^	3 of 4
Insecurity regarding ability to satisfy the partner	**2.14** a	**1.41**	**32 (39)** c	**46 (43)** c	**3 of 4**
Partner response to changes in sexual functioning: accepting/rejecting	2.44^a^	1.77	52 (63)^c^	59 (55)^c^	3 of 4
Level of comfort with one's sexuality	2.20^a^	1.92	36 (43)^c^	49 (46)^c^	3 of 4
Change in the presence of sexual fantasies	1.18	1.00	4 (5)	24 (22)	0 of 4
Level of sexual enjoyment	**2.25** a	**1.92**	**28 (34)** c	**48 (45)** c	**3 of 4**
Sexual satisfaction	**2.56** a	**2.01** b	**53 (64)** c	**62 (58)** c	**4 of 4**
Reduced sexual enjoyment	1.90	1.55	17 (20)	41 (38)^c^	1 of 4
To what extent are sexual dysfunctions distressing	2.43^a^	1.43	50 (60)^c^	48 (45)^c^	3 of 4
Need for care because of sexual difficulties	1.94	1.06	26 (31)^c^	33 (31)^c^	2 of 4
Communication about sexual issues with health professionals	**2.42** a	**1.33**	**59 (71)** c	**33 (31)** c	**3 of 4**
Masturbation^a^	2.02^a^	0.88	22 (27)	19 (18)	
Male sexual health	*N* = 41	*N* = 83	*N* = 83	*N* = 83	
Dry orgasm	1.85	0.83	31 (37)^c^	11 (27)	1 of 4
Retrograde ejaculation	1.83	0.81	25 (30)^c^	6 (15)	1 of 4
Ability to get an erection	2.70^a^	1.82	57 (69)^c^	29 (71)^c^	3 of 4
Ability to maintain an erection (firm enough for sex)	2.67^a^	1.90	52 (63)^c^	32 (78)^c^	3 of 4
Satisfaction with ability to maintain erection or level of firmness	2.57^a^	1.81	50 (60)^c^	21 (51)^c^	3 of 4
Level of confidence in getting an erection and keeping one	**2.39** a	**2.00** b	**42 (51)** c	**28 (68)** c	**4 of 4**
Change in masculinity/feeling less masculine	**2.29** a	**1.24**	**47 (57)** c	**21 (51)** c	**3 of 4**
Female sexual health	*N* = 66	*N* = 83	*N* = 83	*N* = 83	
Insufficient/decreased lubrication	**2.59** a	**1.74**	**60 (72)** c	**43 (65)** c	**3 of 4**
Frequency of spotting/bleeding after sexual intercourse	2.03^a^	1.03	35 (42)^c^	24 (36)^c^	3 of 4
Change in femininity/feeling less feminine	**2.53** a	**1.50**	**63 (76)** c	**46 (69)** c	**3 of 4**

Bold print indicates final issue inclusion; Results are based on descriptive statistics.

a HCP ratings: mean ≥ 2.

b Patient ratings: mean ≥ 2.

HCP ratings ≥30%.

c Patient ratings ≥ 30%.

d Excluded by content redundancy or behavioral norms.

### Phase 2—Construction of the item list: the provisional EORTC SHQ‐22

The 22 SH‐related issues carried forward from phase 1 were operationalized into items 19. Following the model of other EORTC modules, we defined a time frame of 4 weeks. A total of 5 items were retrieved from the EORTC item library 24; 17 new items were developed.

The provisional questionnaire was subsequently translated into nine languages (Croatian, Dutch, French, German, Greek, Italian, Mandarin, Polish, and Spanish).

### Phase 3—Pretesting of the provisional EORTC SHQ‐22

The provisional EORTC SHQ‐22 was pretested in a sample of 171 patients (12 countries). Almost 60% of participants were female; more than two‐thirds (71%) of the patients were currently undergoing treatment and 10% were survivors; about 30% of patients were not sexually active; for details, see Tables 1 and 2.

Quantitative analysis revealed the following results: The criterion mean scores above 1.5 were met for all items but two (incontinence, pain during sexual activity). All items had a range >2 points. All responses in categories 3 and 4 or 1 and 2 were >10% except for item 6 (incontinence). Prevalence of scores 3 or 4 was >50% in 17 items. The remaining five items, all relating to sexual activity, did not fulfill this criterion. The compliance rate was at least 90% for 16 items. The other items with a lower compliance rate related to sexual activity, to having a partner, and to communication with HCP.

In qualitative patient interviews, we recorded more than 120 comments. Item intrusiveness was identified by only nine patients. A total of 30 patients (17.5%) considered some items to be of minor relevance or felt uncomfortable answering the items satisfaction with level of sexual desire, sexual activity, ability to reach an orgasm, and femininity/masculinity. Some items were rated difficult to answer by six patients, and upsetting or confusing by eleven patients. Overall, 23 (13.5%) patients raised additional issues, for example changed body image or changes of body‐ and self‐image (*n* = 4), and masturbation (*n* = 3). A total of 22 patients considered being currently sexually active or having a partnership as an important a priory condition for questionnaire completion.

Interesting to note, the rate of refusal to participate in the study was exceptionally high in Poland (46%; patient age range: 60–67 years). Higher acceptance rates were found (75%) when patients between 42 and 47 years of age were interviewed in an anonymous setting.

Based on quantitative and qualitative results, we adapted or rephrased the items incontinence, pain during sexual activity, sexual activity, decreased libido, ability to reach an orgasm, satisfaction with level of intimacy, partner communication, and communication with HCP; for example, item 14 “Have you been satisfied with your level of affection or intimacy?” was changed to “Have you been satisfied with your level of intimacy?” because patients challenged the use of two different subjects within one question. No items were deleted (see Table 4) or added. A distinct proportion of patients experienced the questionnaire to predominately refer to sexually active patients or patients in a romantic partnership. For others, items did not explicitly relate to their treatment. As a consequence, item order was changed toward grouped sections comprising sexual activity, treatment‐related questions, partner‐related questions, general questions of SH, and sex‐specific questions.

**Table 4:** Results of patient interviews—items after phase 3

Item No.	Item	Mean score (1 not at all – 4 very much)	Scoring 3 (quite a bit) or 4 (very much)	Missing data	Decision^a^
1	Importance of sex life	2.8	69%	4 (2%)	Revised
2	Distress by decreased libido^a^	2.0	71%	4 (2%)	Revised
3	Satisfaction with sexual desire	2.5	52%	11 (6%)	Unchanged
4	Sexual activity was enjoyable	2.7	60%	16 (9%)	Unchanged
5	Satisfaction with the ability to reach an orgasm	2.5	49%^b^	17 (10%)	Unchanged
6	Incontinence (urine/stool)	1.1^a^	98%	18 (10%)^c^	Revised
7	Fatigue or a lack of energy affected sex life	2.4	54%	10 (6%)	Unchanged
8	Cancer treatment affected sexual activity	2.7	42%^b^	12 (7%)	Unchanged
9	Pain during/after sexual activity	1.5^a^	87%	21 (12%)^c^	Unchanged
10	Worries that sex would be painful	1.9	75%	17 (10%)	Unchanged
11	Satisfaction with communication with health professionals	2.4	53%	31 (18%)^c^	Revised
12	Satisfaction with communication (partner)	3.1	75%	19 (11%)^c^	Revised
13	Worries that their partner may cause them pain during sexual contact	1.7	82%	14 (8%)	Unchanged
14	Satisfaction with affection or intimacy	2.8	64%	11 (6%)	Revised
15	Insecure regarding the ability to satisfy their partner	2.1	64%	17 (10%)	Unchanged
16	Sexual activity	2.1	34%^b^	4 (2%)	Revised
17	Extent of their sexual enjoyment	2.3	47%^b^	11 (6%)	Unchanged
18	Satisfaction with sex life	2.4	53%	10 (6%)	Unchanged
19	*Male patients only (N = 70)* **:** Confidence in an erection when having sex	2.3	37%^b^	7 (10%)^c^	Unchanged
20	Feeling less masculine	1.9	72%	3 (4%)	Unchanged
21	*Female patients only (N = 101)*: Dry vagina	2.3	60%	11 (11%)^c^	Unchanged
22	Feeling less feminine	2.3	60%	2 (2%)	Unchanged

Based on the criteria for item retention.

a Mean score ≤ 1.5

b Scoring 3 or 4 ≤ 50%.

c Compliance rate < 90%.

Results are based on descriptive statistics.

#### Psychometric properties

The exploratory factor analysis (principal component analysis) suggested a two‐factor solution (sexual satisfaction and sexual pain) and 11 single items accounting for 65.4% of the variance. Both scales showed good internal consistency indicated by Cronbach's alpha of 0.87 and 0.82 (see Table 5) as well as good item‐scale correlations of 0.7**–**0.8 for sexual satisfaction and 0.8–0.9 for sexual pain. The factor “sexual satisfaction” includes issues related to sexual activity, sexual enjoyment, sexual functioning, and intimacy, which will be further explored in phase 4.

**Table 5:** Results of the exploratory factor analysis

	Item No.	Items	M	SD	Cronbach's Alpha^2^	Item‐scale correlation	*N*
Factor 1 Sexual satisfaction 8 items)	16	Sexual activity	50.33	25.77	0.87	0.7	165
	3	Satisfaction with sexual desire				0.7	
	4	Sexual activity was enjoyable				0.8	
	5	Satisfaction with the ability to reach an orgasm				0.8	
	11	Satisfaction with affection or intimacy				0.7	
	12	Satisfaction with communication (partner)				0.6	
	18	Satisfaction with sex life				0.8	
	17	Extent of their sexual enjoyment				0.8	
Factor 2 Sexual Pain (3 items)	9	Pain during/after sexual activity^a^	77.04	28.06	0.82	0.8	159
	10	Worries that sex would be painful^a^				0.9	
	13	Worries that their partner may cause them pain during sexual contact^a^				0.9	
11 Single items	1	Importance of sex life	43.40	39.29		–	159
	2	Distress by decreased libido^a^	61.28	30.68		–	167
	6	Incontinence (urine/stool)^a^	63.20	35.99		–	154
	7	Fatigue or a lack of energy affected their sex life^a^	68.26	35.44		–	167
	8	Cancer treatment affected sexual activity^a^	96.30	12.42		–	153
	15	Insecure regarding the ability to satisfy their partner^a^	53.21	39.15		–	161
	11	Satisfaction with communication (health professionals)	48.68	37.91		–	139
	19	Confidence in an erection when having sex	42.86	39.00		–	63
	20	Feeling less masculine^a^	71.72	38.45		–	66
	21	Dry vagina^a^	57.47	36.55		–	87
	22	Feeling less feminine^a^	57.39	35.27			97

a Derived by an explorative principal component analysis with the oblique rotation method (promax rotation).

Determination of the scale reliability by means of Cronbach's alpha for internal consistency.

## Discussion

To the best of our knowledge, the EORTC SHQ‐22 is unique in terms of conceptualization. We followed the WHO definition of SH in order to construct an instrument covering both, sexual functioning (including symptoms and treatment side effects) and a psychosexual component. This integrative approach has previously been demonstrated to be of vital importance in cancer care 14; this assumption was confirmed by our phase 1 results. Patients identified several issues beyond sexual functioning such as sexual satisfaction or partner communication as highly relevant. Of note, issues relating to frequency or norms of sexual activity were removed as experienced least relevant. The questionnaire is further designed to be used in survivors as sexual impairments persist into survivorship 25, 26. Patient and HCP feedback underscored the relevance of SH problems in the survivorship phase and did not suggest a difference on‐ and off‐treatment. Only incontinence and pain during intercourse seemed to be relevant only for subgroups of patients, which can be regarded as a questionnaire's limitation. However, this approach has been followed by other EORTC questionnaires, for example the testicular cancer module, the EORTC QLQ‐TC26 (Holzner et al., 2013). However, we have to acknowledge that the questionnaire does not address age‐dependent issues.

The substantial sample size in phase 3 facilitated comprehensive, well‐balanced patient feedback across countries and cancer sites. According to patient feedback, the items seem to cover the full range of SH issues, are well understood, and not intrusive, except in Poland.

The previously reported patient reluctance to answer specific SH questions was reflected also in this study by an unsatisfactory compliance rate for some items 27. Although this is an expected result as sexuality is well known to be an inherently sensitive subject, we have to acknowledge this limitation 28, 29. Here, it was the items on sexual activity and communication that received lower compliance rates. A possible explanation is that for some patients, sexual activity is exclusively conceptualized as a partner‐related activity (i.e., masturbation is not considered). Therefore, some patients may not have considered themselves to be sexually active unless they physically engaged in sexual activity with a partner, contributing to the 30% of respondents who classified themselves as not sexually active in this sample.

The preliminary, explorative psychometric analysis revealed two factors with good internal consistency above 0.8 and numerous single items. In view of these empiric results in phase 3, there seems to be an emphasis on sexual functioning at least in terms of the factor solution. Psychosexual and social issues are however covered by single items so that the integrative approach for the measurement of sexual health is still reflected. Preliminary psychometric results need further confirmation in the final phase 4 validation study, which will incorporate confirmatory factor analysis. In addition, sample size restricted providing results for subgroup comparisons on sexual health outcome, for example age or cancer sites and cancer stages, which would have been interesting in terms of applicability. This is true in particular also for the groups of sexually active versus inactive patients. However, these analyses are provided in phase 4.

As indicated, we observed a good cross‐cultural acceptance. One country‐specific result was noted: In Poland, the rejection rate of patients approached for the study was distinctly higher compared to other countries. At this point, we can only hypothesize on related reasons as the protocol did not include the assessment of reasons for nonparticipation. From the qualitative debriefing interviews with patients and HCP, we gathered evidence of the impact of culture on the understanding of sexuality 30, 31. We identified two Polish culture‐specific factors, namely a high rate of very religious people and the upbringing of Polish (older) people making sexuality a taboo subject 32. Sexuality as a taboo subject seems to have a long tradition in Poland 30.

## Conclusions

The EORTC SHQ‐22 as a stand‐alone, multidimensional QOL instrument to measure sexual health in patients with cancer is clinically applicable, covers relevant SH issues, and shows high acceptability across the heterogeneous target population. The final development phase, that is psychometric validation (phase 4) including large‐scale, cross‐cultural field testing of the EORTC SHQ‐22, is ongoing.

## Conflict of Interest

None.

## Informed Consent

Informed consent was obtained from all individual participants included in the study.

## Ethical Approval

All procedures performed in studies involving human participants were in accordance with the ethical standards of the institutional and/or national research committees and with the 1964 Helsinki declaration and its later amendments or comparable ethical standards.

## References

[1] Ananth, H. , L. Jones , M. King , and A. Tookman . 2003 The impact of cancer on sexual function: a controlled study. Palliat. Med. 17:202–205. 12701852 10.1191/0269216303pm759oa

[2] Krychman, M. L. , L. Pereira , J. Carter , and A. Amsterdam . 2006 Sexual oncology: sexual health issues in women with cancer. Oncology 71:18–25. 17347586 10.1159/000100521

[3] Mulhall, J. P. , L. Incrocci , I. Goldstein , and R. Rosen . 2011 Cancer and sexual health: current clinical urology. Springer Science, New York.

[4] National Health Institute . 2006 The prevalence and types of sexual dysfunction in people with cancer. In.

[5] Huffman, L. B. , E. M. Hartenbach , J. Carter , J. K. Rash , and D. M. Kushner . 2016 Maintaining sexual health throughout gynecologic cancer survivorship: a comprehensive review and clinical guide. Gynecol. Oncol. 140:359–368. 26556768 10.1016/j.ygyno.2015.11.010 PMC4835814

[6] Hummel, S. B. , D. E. E. Hahn , J. van Lankveld , H. S. A. Oldenburg , E. Broomans , and N. K. Aaronson . 2017 Factors associated with specific diagnostic and statistical manual of mental disorders, fourth edition sexual dysfunctions in breast cancer survivors: a study of patients and their partners. J. Sex Med. 14:1248–1259. 28923310 10.1016/j.jsxm.2017.08.004

[7] Whicker, M. , J. Black , G. Altwerger , G. Menderes , J. Feinberg , and E. Ratner . 2017 Management of sexuality, intimacy, and menopause symptoms in patients with ovarian cancer. Am. J. Obstet. Gynecol. 217:395–403. 28411144 10.1016/j.ajog.2017.04.012

[8] Bertero, C. , and M. Chamberlain Wilmoth . 2007 Breast cancer diagnosis and its treatment affecting the self: a meta‐synthesis. Cancer Nurs. 30:194–202. quiz 203‐4. 17510582 10.1097/01.NCC.0000270707.80037.4c

[9] Gilbert, E. , J. M. Ussher , and J. Perz . 2010 Sexuality after breast cancer: a review. Maturitas 66:397–407. 20439140 10.1016/j.maturitas.2010.03.027

[10] Ussher, J. M. , J. Perz , and E. Gilbert . 2015 Australian Cancer Sexuality Study Team. Perceived causes and consequences of sexual changes after cancer for women and men: a mixed method study. BMC Cancer 15:268. 25885443 10.1186/s12885-015-1243-8 PMC4407322

[11] Cull, A. M. 1992 The assessment of sexual function in cancer patients. Eur. J. Cancer 28A:1680–1686. 1389485 10.1016/0959-8049(92)90068-d

[12] Jeffery, D. D. , J. P. Tzeng , F. J. Keefe , L. S. Porter , E. A. Hahn , K. E. Flynn , et al. 2009 Initial report of the cancer Patient‐Reported Outcomes Measurement Information System (PROMIS) sexual function committee: review of sexual function measures and domains used in oncology. Cancer 115:1142–1153. 19195044 10.1002/cncr.24134 PMC2742328

[13] WHO . 2012 Sexual health. In.

[14] Cleary, V. , and J. Hegarty . 2011 Understanding sexuality in women with gynaecological cancer. Eur. J. Oncol. Nurs. 15:38–45. 20584629 10.1016/j.ejon.2010.05.008

[15] DeRogatis, L. R. 2008 Assessment of sexual function/dysfunction via patient reported outcomes. Int. J. Impot. Res. 20:35–44. 17703221 10.1038/sj.ijir.3901591

[16] Hoole, J. , A. Kanatas , A. Calvert , S. N. Rogers , A. B. Smith , and D. A. Mitchell . 2015 Validated questionnaires on intimacy in patients who have had cancer. Br. J. Oral Maxillofac. Surg. 53:584–593. 26037739 10.1016/j.bjoms.2015.05.003

[17] White, I. D. , A. Sangha , G. Lucas , and T. Wiseman . 2016 Assessment of sexual difficulties associated with multi‐modal treatment for cervical or endometrial cancer: a systematic review of measurement instruments. Gynecol. Oncol. 143:664–673. 27671030 10.1016/j.ygyno.2016.08.332

[18] Bartula, I. , and K. A. Sherman . 2013 Screening for sexual dysfunction in women diagnosed with breast cancer: systematic review and recommendations. Breast Cancer Res. Treat. 141:173–185. 24013707 10.1007/s10549-013-2685-9 PMC3824351

[19] European Organization for Research and Treatment of Cancer – Quality of life group . 2011 Guidelines for developing questionnaire modules. In.

[20] Moreira, E. D. Jr , G. Brock , D. B. Glasser , A. Nicolosi , E. O. Laumann , A. Paik , et al. 2005 Help‐seeking behaviour for sexual problems: the global study of sexual attitudes and behaviors. Int. J. Clin. Pract. 59:6–16. 15707457 10.1111/j.1742-1241.2005.00382.x

[21] Emery, M. P. , L. L. Perrier , and C. Acquadro . 2005 Patient‐reported outcome and quality of life instruments database (PROQOLID): frequently asked questions. Health Qual. Life Outcomes 3:12. 15755325 10.1186/1477-7525-3-12 PMC555954

[22] European Organization for Research and Treatment of Cancer – Quality of Life Group . Item library. In.

[23] IBM . SPSS statistics for windows, version 22.0. In. IBM Corp., Armonk, NY; released 2013.

[24] European Organization for Research and Treatment of Cancer – Quality of Life Group . Questionnaires. In.

[25] Bober, S. L. , and V. S. Varela . 2012 Sexuality in adult cancer survivors: challenges and intervention. J. Clin. Oncol. 30:3712–3719. 23008322 10.1200/JCO.2012.41.7915

[26] Dizon, D. S. 2009 Quality of life after breast cancer: survivorship and sexuality. Breast J. 15:500–504. 19614908 10.1111/j.1524-4741.2009.00766.x

[27] Carr, S. 2007 Talking about sex to oncologists and about cancer to sexologists. Sexologies 16:267–272. Elsevier.

[28] McCoy . 2000 The McCoy female sexuality questionnaire. Qual. Life Res. 9:739–745.

[29] Hordern, A. J. , and A. F. Street . 2007 Constructions of sexuality and intimacy after cancer: patient and health professional perspectives. Soc. Sci. Med. 64:1704–1718. 17261346 10.1016/j.socscimed.2006.12.012

[30] Martyniuk, U. , A. Dekker , S. Sehner , H. Richter‐Appelt , and P. Briken . 2015 Religiosity, sexual myths, sex taboos, and pornography use: a cross‐national comparison of Polish and German university students. J. Psychosocial Res. Cyberspace 9(2), article 4. doi: 10.5817/CP2015-2-4.

[31] Agocha, V. B. , M. Asencio , and C. U. Decena . 2014 Sexuality and culture Pp. 183–228 in Association A. P., ed. APA handbook of sexuality and psychology. American Psychological Association , Washington, DC.

[32] CBOS Poland . 2012 Changing religiosity of the poles. Pol. Public Op. 4:1–4.

